# Hair Loss After Metabolic and Bariatric Surgery: a Systematic Review and Meta-analysis

**DOI:** 10.1007/s11695-021-05311-2

**Published:** 2021-03-05

**Authors:** Wen Zhang, Meiling Fan, Cunchuan Wang, Kamal Mahawar, Chetan Parmar, Weiju Chen, Wah Yang, on behalf of Global Bariatric Research Collaborative

**Affiliations:** 1 School of Nursing, Jinan University, Guangzhou, No.601, Huangpu Avenue West, Guangzhou, Guangdong China; 2 Department of Biliary Tract Surgery, West China Hospital, Sichuan University, Chengdu, Sichuan China; 3 Department of Metabolic and Bariatric Surgery, The First Affiliated Hospital, Jinan University, No. 613, Huangpu Avenue West, Guangzhou, Guangdong China; 4 Joint Institute of Metabolic Medicine between State Key Laboratory of Pharmaceutical Biotechnology, The University of Hong Kong and Jinan University, Guangzhou, China; 5 Bariatric Unit, Sunderland Royal Hospital, Sunderland, UK; 6 Department of Surgery, Whittington Hospital, London, UK; 7 University College London Medical School, London, UK; 8 State Key Laboratory of Pharmaceutical Biotechnology, LKS Faculty of Medicine, The University of Hong Kong, Hong Kong, China; 9 Department of Medicine, LKS Faculty of Medicine, The University of Hong Kong, Hong Kong, China

**Keywords:** Hair loss, Bariatric surgery, Metabolic surgery, Nutrition, Meta-analysis

## Abstract

**Background:**

Hair loss is a common complication after metabolic and bariatric surgery (MBS). There is a lack of published systematic review in the scientific literature on this topic. The aim of this study was to perform a systematic review and meta-analysis on hair loss after MBS in accordance with Preferred Reporting Items for Systematic reviews and Meta-Analysis (PRISMA) guidelines.

**Methods:**

PubMed, CINAHL, EMBASE, Web of Science, SCOPUS, and four Chinese databases were searched. Data were pooled using Review Manager 5.3 and Stata 12.0, and subgroups were performed if necessary and feasible.

**Results:**

A total of 18 studies (*n* = 2538) were included. The pooled results showed that the incidence of hair loss after MBS was 57% (95% CI 42–71%). It decreased with longer follow-up times. Hair loss was significantly more common in younger (mean difference (MD), − 2.45; 95% CI, − 4.26 to − 0.64; *p* = 0.008) women (OR, 3.87; 95% CI, 0.59 to 17.59; *p* = 0.08). Serum zinc (standardized mean difference (SMD), − 1.13; 95% CI, − 2.27 to 0.01, *p* = 0.05), folic acid (SMD = − 0.88, 95% CI − 1.29 to − 0.46, *p* < 0.0001), and ferritin levels (SMD, − 0.22; 95% CI, − 0.38 to − 0.05; *p* = 0.01), but not serum iron and vitamin B_12_, were associated with hair loss following MBS.

**Conclusions:**

Hair loss is common after MBS especially in younger women, and those with low serum levels of zinc, folic acid, and ferritin. Prospective studies on larger cohorts are needed.

## Introduction

The prevalence of overweight and obesity has been increasing globally and it has become a major public health concern in many countries [1]. Metabolic and bariatric surgery (MBS) is now a recognized treatment strategy for patients with severe, complex obesity meeting the accepted criteria [2, 3].

Hair loss is a recognized complication of MBS. Some researchers have noted hair loss in more than half of the patients in the short term after MBS [4]. Others have demonstrated that iron and zinc levels are associated with hair loss [5]. Although hair loss does not usually result in severe morbidity, it can cause unnecessary alarm and affect mental health, self-esteem, and quality of life of the patient [6].

The incidence of hair loss after MBS varies in different studies. One study reported that 15.4% of patients experienced hair loss within 3 months after RYGB [7]. A previous study by some of the authors found that the incidences were 55% and 40% after laparoscopic sleeve gastrectomy (LSG) and laparoscopic Roux-en-Y gastric bypass (LRYGB) respectively within 6 months. It is similar to Ruiz-Tovar’s report of 41% after LSG, in 2014 [5]. Others have found the incidence to be as high as 80% after laparoscopic gastric plication (LGP) or LSG [8].

There is some suggestion that the incidence decreases with time. For example, Ledoux et al. [4] reported that the incidence of hair loss after MBS decreased from 65% at ≤ 1 year to 35% at ≥ 3 years. Guo et al. [9] observed that the onset time and end time of hair loss were 3.4 ± 1.4 months and 9.03 ± 3.6 months, respectively. In 42 cases of hair loss, 15 patients received oral medication without significant improvement of hair loss. However, during the follow-up period (15months), hair loss stopped and new hair gradually grew out in all patients. The incidence of short-term and long-term hair loss after bariatric surgery is not consistent.

There is also considerable debate in the scientific literature regarding its etio-pathogenesis. For instance, Katsogridaki et al. [10] found that vitamin B_12_ tended to be lower in patients with hair loss compared with controls. Nevertheless, the opposite conclusion was found by Ledoux et el. [4] Furthermore, Sen et al. [11] also did not find any therapeutic benefit with Biotin supplements. Role of other micronutrient deficiencies such as those of folic acid, zinc, and iron is also controversial [4, 5, 9].

There is currently no published systematic review in the scientific literature on hair loss after MBS examining all these various issues. We, therefore, performed a systematic review and meta-analysis on this topic in accordance with Preferred Reporting Items for Systematic reviews and Meta-Analysis (PRISMA) guidelines [12].

## Methods

Two authors independently and systematically performed literature searches on PubMed, CINAHL, EMBASE, Web of Science, SCOPUS, China National Knowledge Infrastructure (CNKI), the Database of Chinese Ministry of Science & Technology (Wanfang), and the Database of Chinese Science and Technology Periodicals (VIP) from the inception of the database to July 2020, with the following search terms: “(bariatric surgery OR weight loss surgery OR obesity surgery OR metabolic surgery OR gastric bypass OR sleeve gastrectomy OR gastric banding OR duodenojejunal bypass OR duodenal switch) AND (alopecia OR hair loss)”**.** In addition, we also manually searched the reference list of published documents.

### Inclusion and Exclusion Criteria

Articles were included if they met the following criteria: (1) patients with body mass index (BMI) greater than 30 kg/m^2^, age 18–65 years, and (2) provide the incidence of hair loss after MBS or its etiological factors.

Case reports, reviews and non-English/Chinese studies, revision surgery, and conference abstracts of unpublished data were excluded. For studies enrolled overlapping populations, we only included the study with the most comprehensive information.

### Data Extraction and Quality Assessment

Two researchers (Wen Zhang and Meiling Fan) independently screened the title, abstract, and full text of the articles based on the inclusion and exclusion criteria. Any discrepancies that occurred during the full-text screening stage were resolved by consensus between the two reviewers. The characteristics of the included studies are presented in Table 1. A pre-specified data extraction form was used to record the following data: the first author of the study, the year of publication, location of trial, number of subjects, sex, mean age at time of surgery, length of follow-up, mean BMI before surgery, study design, type of bariatric surgery, and outcome indicators.

**Table 1:** Characteristic of included studies

Author (year)	Country	Design	Follow-up (months)	Mean age (years)	Number(M/F)	Procedure	Pre-BMI (kg/m^2^)	Hair loss (%)	Quality assessment
Ledoux 2020 [4]	France	Retrospective	7.4 ± 2.5	43.5 ±18.8	275 (38/237)	RYGB	33.5 ± 9.2	162 (58.9)	8
	France	Retrospective	6.8 ± 2.0	42.1 ± 11.0	280 (40/240)	SG	32.7 ± 5.4	176 (62.9)	
	France	Retrospective	60.5 ± 19.7	48.2 ± 10.7	383 (44/339)	RYGB	31.5 ± 6.2	140 (36.6)	
	France	Retrospective	48.9 ± 17.0	46.3 ± 10.3	111 (11/100)	SG	34.0 ± 8.5	35 (31.5)	
Lin 2019 [13]	China	Retrospective		31.5 ± 9.9	83 (44/39)	SG+JJB	44.7 ± 7.6	55 (66.3)	6
	China	Retrospective	12	26 ± 6.8	82 (41/41)	SG	42.5 ± 6.9	48 (58.5)	
	China	Retrospective		34.8 ± 12.3	79 (37/42)	RYGB	42.4 ± 5.3	62 (78.5)	
Castanha 2018 [14]	Brazil	Cross-sectional	41.87 ± 37.5	44.2 ± 10.8	103 (11/92)	SVG/RYGB	48.1	82 (79.6)	3
Du 2018 [15]	China	Retrospective	≥ 12	47.7 ± 8.6	97 (33/64)	LGB	34.6 ± 5.6	1 (1.0)	7
	China	Retrospective		41.8 ± 10.5	79 (22/57)	LSG	35.4 ± 5.3	0 (0)	
Katsogridaki 2018 [10]	Greece	Prospective	6	38.7 ± 11.9	50 (14/36)	LSG	44.5 ± 6.1	28 (56.0)	7
Trindade 2017 [16]	Brazil	Cross-sectional	≥ 24	20-65	N	RYGB	N	23 (79.3)	3
Goldenshluger 2017 [17]	Israel	Retrospective	36	39.9 ± 11.2	178 (57/121)	LSG	42.9 ± 4.5	76 (42.7)	6
Guo 2017 [9]	China	Retrospective	15	31 (17-43)	54 (11/43)	LSG	35.0 ± 6.0	42 (77.8)	6
Talebpour 2017 [8]	Iran	RCT	24	38.6 ± 10.3	35 (6/29)	LSG	44.6 ± 3.5	28 (80.0)	5
	Iran	RCT	24	35.3 ± 10.1	35 (8/27)	LGP	48.4 ± 4.9	28 (80.0)	
Dagan 2017 [18]	Israel		3					18 (23.4)	5
	Israel	Prospective	6	43.1 ± 9.3	77 (33/44)	LSG	42.1 ± 4.8	39 (50.6)	
	Israel		12					25 (32.5)	
Lee 2016 [19]	Korea	Retrospective	11.6	33.6 ± 10.3	72 (17/55)	LAGB	38.9 ± 5.4	0 (0)	7
	Korea	Retrospective	7.0	39.1 ± 11.1	73 (17/56)	RYGB	39.0 ± 6.9	1 (1.4)	
	Korea	Retrospective	6.3	35.0 ± 10.4	116 (30/86)	SG	39.1 ± 6.2	0 (0)	
Santos 2015 [20]	Brazil	Retrospective	7	40.1 ± 11.8	61 (0/61)	RYGB	46.0 ± 6.1	27 (44.7)	4
			12					16 (26.2)	
Barros 2015 [21]	Brazil	Cross-sectional study	7-24	40.53 ± 10.03	92 (16/76)	RYGB	47.2 ± 6.8	46 (74.2)	4
Silva 2014 [22]	Brazil	Cross-sectional study	≥ 1	18-59	70 (13/57)	RYGB	N	44 (62.9)	3
Pan 2014 [23]	China	Cross-sectional study	≥ 12	31.2 ± 9.9	82 (33/49)	LSG	41.79 ± 6.88	15 (18.3)	4
Ruiz-Tovar 2014 [5]	Spain	Prospective	12	44.2 ± 10.4	42 (0/42)	LSG	51.2 ± 7.8	17 (40.5)	7
Boza 2011 [24]	Chile	Retrospective	1–72	37.8 ± 12.4	199 (59/140)	LAGB	36.0 ± 3.8	9 (4.5)	5
Depaula 2011 [25]	Brazil	Retrospective	25–61	41.4 ± 12.1	120 (49/71)	LII-SG	43.4 ± 4.2	94 (79.7)	7

*RYGB* Roux-en-Y gastric bypass, *SG* sleeve gastrectomy, *SG+JJB* sleeve gastrectomy with jejunojejunal bypass, *LSG* laparoscopic sleeve gastrectomy, *LGB* laparoscopic gastric banding, *LII-SG* laparoscopic ileal interposition associated with a sleeve gastrectomy, *LGP* laparoscopic gastric placation, *LAGB* laparoscopic adjustable gastric banding, *SVG* sleeve vertical gastrectomy, *BMI* body mass index

To evaluate the quality of the observational studies [4, 5, 9, 10, 13, 15, 17–20, 24, 25], we employed the nine-point Newcastle-Ottawa Scale (NOS) [26], which assesses three fundamental aspects of methodology: study participant selection (0–4 points), confounder adjustment (0–2), and outcome indicator determination (0–3). A study with an NOS score of 7–9 points was defined as high quality. The methodological quality of the cross-sectional studies [14, 16, 21–23] included was assessed using an 11-item checklist recommended by Agency for Healthcare Research and Quality (AHRQ) [27]. An item would be scored “0” if it was answered “NO” or “UNCLEAR”; if it was answered “YES,” then the item scored “1.” Article quality was assessed as follows: low quality = 0–3; moderate quality = 4–7; high quality = 8–11. The Talebpour study in 2018 was a randomized controlled trial (RCT) [8] and was assessed using the Jadad scale [28]. A study with a Jadad score of 4 or more was considered to be high quality. The quality score is presented in Table 1.

### Statistical Analysis

Categorical variables are reported as frequencies and percentages, and continuous data are expressed as the mean ± standard deviation (SD). For continuous variables, the mean difference (MD) or standardized mean difference (SMD) with the 95% confidence interval (CI) was used when appropriate, depending on whether or not the outcomes were measured by the same scales, while the odds ratio (OR) with 95% CI was used for dichotomous variables (hair loss frequency). All statistical analyses were performed on Review Manager (RevMan version 5.3) and Stata (version 12.0), with the significance level set to *p* < 0.1. Heterogeneity was measured using the I-square statistic, with a significance threshold of I^2^>50% [29]. A random effects model was used if the *I*^2^ statistic was significant; otherwise, a fixed effects model was used. Pre-specified subgroup analyses based on follow-up duration (≥ 12 months vs. < 12 months), because studies suggested that the hair loss rate would decrease along with longer follow-up times. In addition, we performed a subgroup analysis based on the two most common procedures (RYGB vs. SG). Meta-analysis results are expressed using forest plots.

## Results

### Literature Retrieval Results and Basic Characteristics

**Fig. 1:**
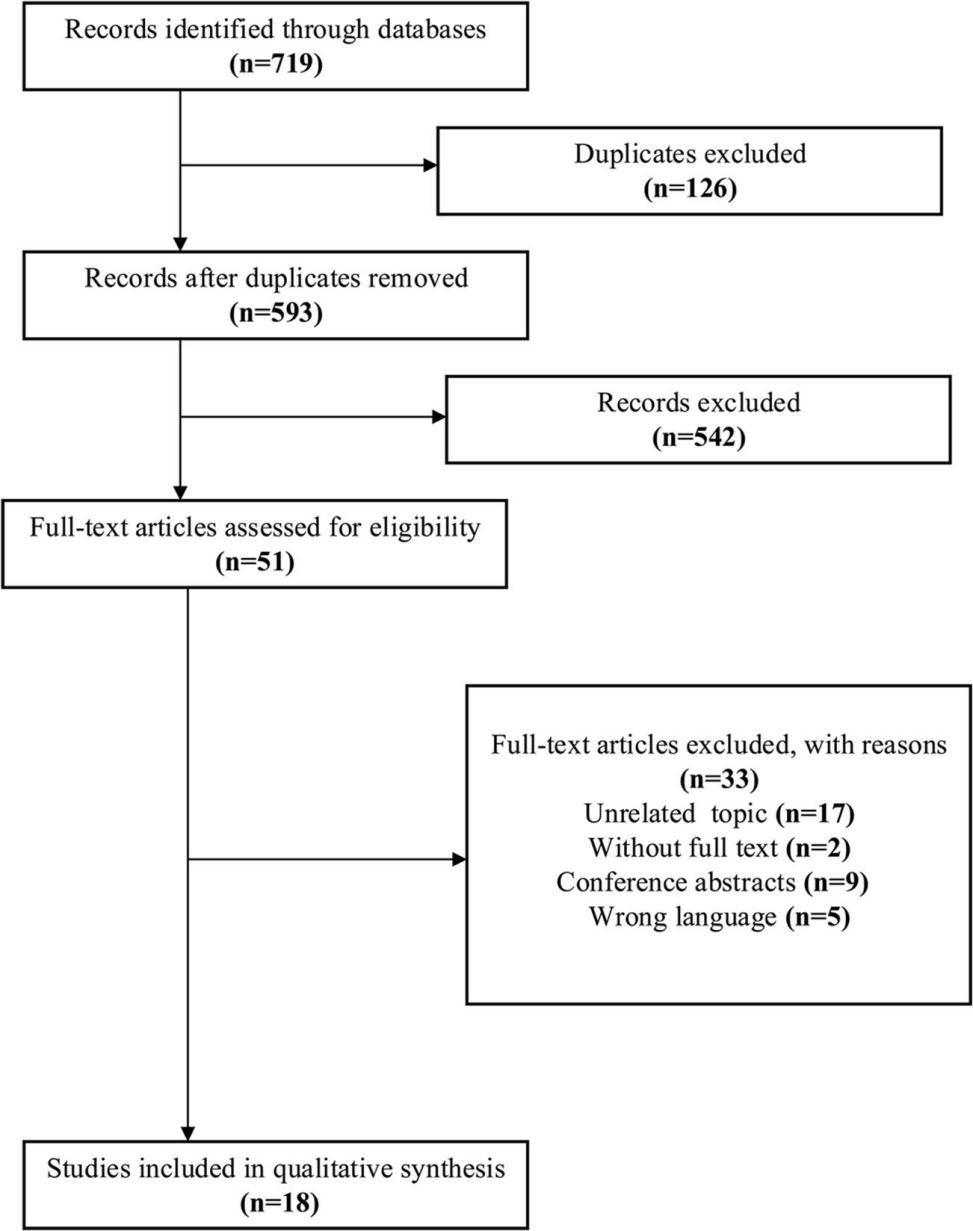
Flow diagram of study selection

Figure 1 shows the study selection flowchart. Through the literature search, we identified 719 citations. Using the EndNote X9 software for document management, we removed any duplicates and were left with 593 references. After excluding irrelevant reports by reviewing titles and abstracts, we then retrieved 51 full-text articles that were eligible. There were 17 articles with unrelated topics, 2 without full text, 9 conference abstracts, and 5 non-English/Chinese literatures excluded. Ultimately, 18 original articles were included, as shown in the study flowchart.

### Study Characteristics and Quality Assessment

The study characteristics are presented in Table 1. We identified 18 eligible studies (*N* = 2538) [4, 5, 8–10, 13–25] that were published from 2011–2020. These studies included 1 RCT [8], 3 prospective observational studies [5, 10, 18], 8 retrospective observational studies [4, 9, 13, 15, 17, 19, 20, 25], and 5 cross-sectional studies [13, 14, 21–23]. The duration of follow-up ranges from 1 to 72 months. Four studies analyzed the influencing factors of hair loss after MBS [4, 5, 9, 10]. Two studies were published in Chinese [9, 23]; the others were available in English.

According to the Newcastle-Ottawa Scale and AHRQ, the quality of observational studies was Low or moderate. The RCT, with a score of 5 on the Jadad scale, was a high-quality study. The assessment of study quality is displayed in Table 1.

### Meta-analysis of Incidence of Hair Loss After MBS

**Fig. 2:**
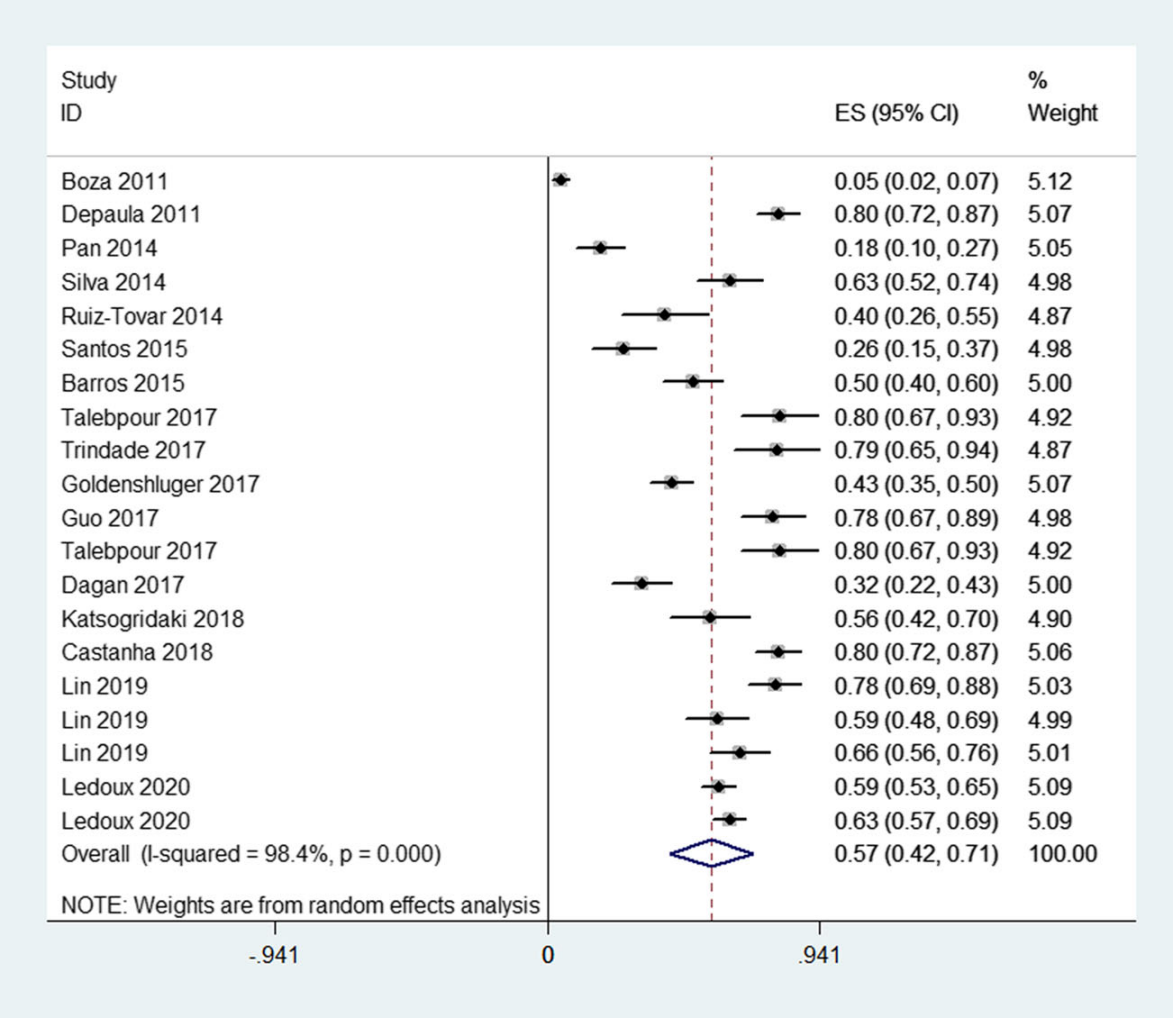
Forest plots of overall incidence

In this review, 18 articles [4, 5, 8–10, 13–25] including a total of 2538 patients were included. The overall incidence of hair loss ranged from 4.5% to 80%. Because of significant heterogeneity among these studies (*p* = 0.000, *I*^2^ = 98.4%) (Fig. 2), a random effects model was used to pool the results. The results showed that the pooled incidence of hair loss was 57% (95%CI, 42–71%). The Egger (*p* = 0.005) test showed that there was significant publication bias in the literature. This may be due to the low quality of the included studies.

### Subgroup Analysis

**Fig. 3:**
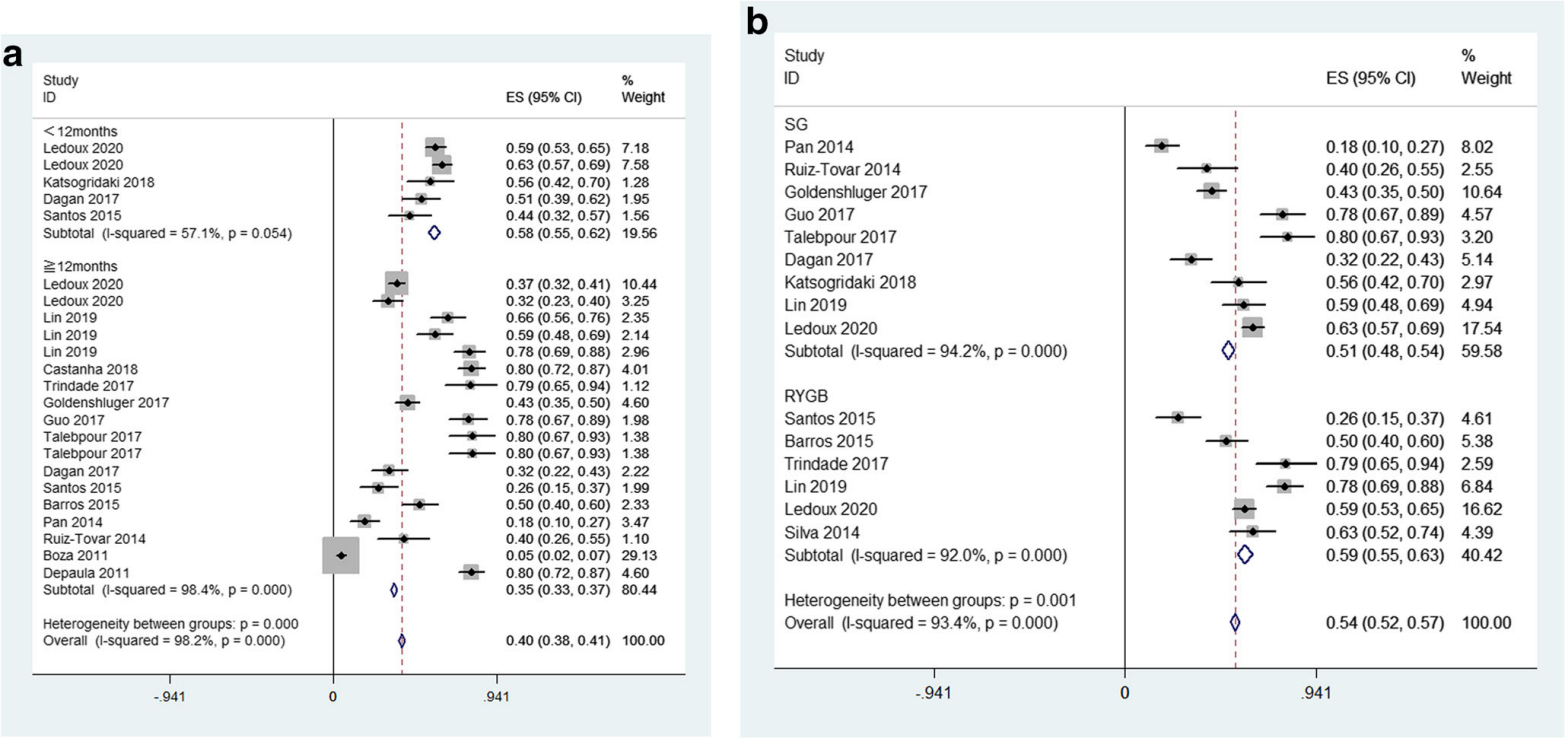
**a** Forest plots of follow-up duration. **b** Forest plots of procedures

We performed subgroup analyses by follow-up duration (≥ 12 months vs. < 12 months). When we looked at the subgroup based on follow-up duration, a significant pooled result (35%, 95% CI 33 to 37%, *p*<0.1) was observed for the articles with long-term follow-up (LT ≥ 12 months), but not for the studies with short-term follow-up (ST < 12 months) (58%, 95% CI 55 to 62%, *p* = 0.054). We found that the incidence of hair loss decreased with longer follow-up times, which decreased from58 (ST) to 35% (LT) (Fig. 3a).

In addition, we conducted a subgroup analysis based on the two most common procedures (SG vs RYGB) included in the study. When we looked at the subgroup of procedures, both the two subgroups had significant pooled results, but the incidence of hair loss was similar in both groups, with SG surgery (51%, 95% CI 48 to 54%, *p*< 0.1, *I*^2^ = 94.2%) and RYGB surgery (59%, 95% CI 55 to 63%, *p*<0.1, *I*^2^ = 92.0%) ( Fig. 3b).

### Meta-analysis of Factors Influencing Hair Loss After MBS

Five studies analyzed the factors related to hair loss after MBS [4, 5, 9, 10, 23]. A total of 783 patients were included, including 419 hair loss patients and 364 controls. Because the number of included studies was less than 10, publication bias was not checked for this outcome. We did not perform subgroup analyses.

**Fig. 4:**
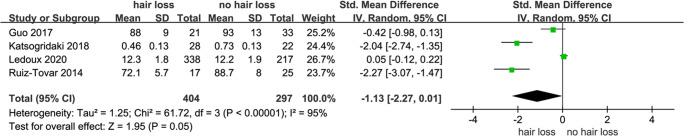
Forest plots of studies in serum zinc

### Zinc

Four studies were included in the meta-analysis of zinc [4, 5, 9, 10], with 701 patients overall (Table 2). Because of heterogeneity in included studies (*p* =0.05, *I*^2^ =95%) (Fig. 4), a random effects model was chosen to pool results. It showed that zinc concentration was lower in patients with hair loss after MBS (SMD, − 1.13; 95% CI, − 2.27 to 0.01, *p* = 0.05) with the follow-up time was 6.8 months to 15 months.

**Table 2:** Studies of zinc and hair loss

Author (year)	Demographics (hair loss group)	Measures and outcomes
Ledoux 2020 [4]	Sex: 10M/328F Age: 41.5 ± 10.2 Country: France	No difference in serum zinc levels in hair loss patients (12.3 ± 1.8 μmol/l) vs. controls (12.2 ± 1.9 μmol/l)
Katsogridaki 2018 [10]	Sex: 5M/23F Age: 38.54 ± 11.04 Country: Greece	Mean zinc levels in hair loss patients (0.46 ± 0.13 mcg/ml) vs controls (0.73 ± 0.13 mcg/ml) (*p* < 0.01)
Guo 2017 [9]	Sex: 1M/20F Age: 31 ± 6 Country: China	No difference in serum zinc levels in hair loss patients (88 ± 9 μmol/l) vs. controls (93 ± 13 μmol/l)
Ruiz-Tovar 2014 [5]	Sex: 0F/16F Country: Spain	Mean zinc levels in hair loss patients (72.1 ± 5.7 mg/dL) vs controls (88.7 ± 8 mg/dL) (*p* = 0.021)

**Fig. 5:**
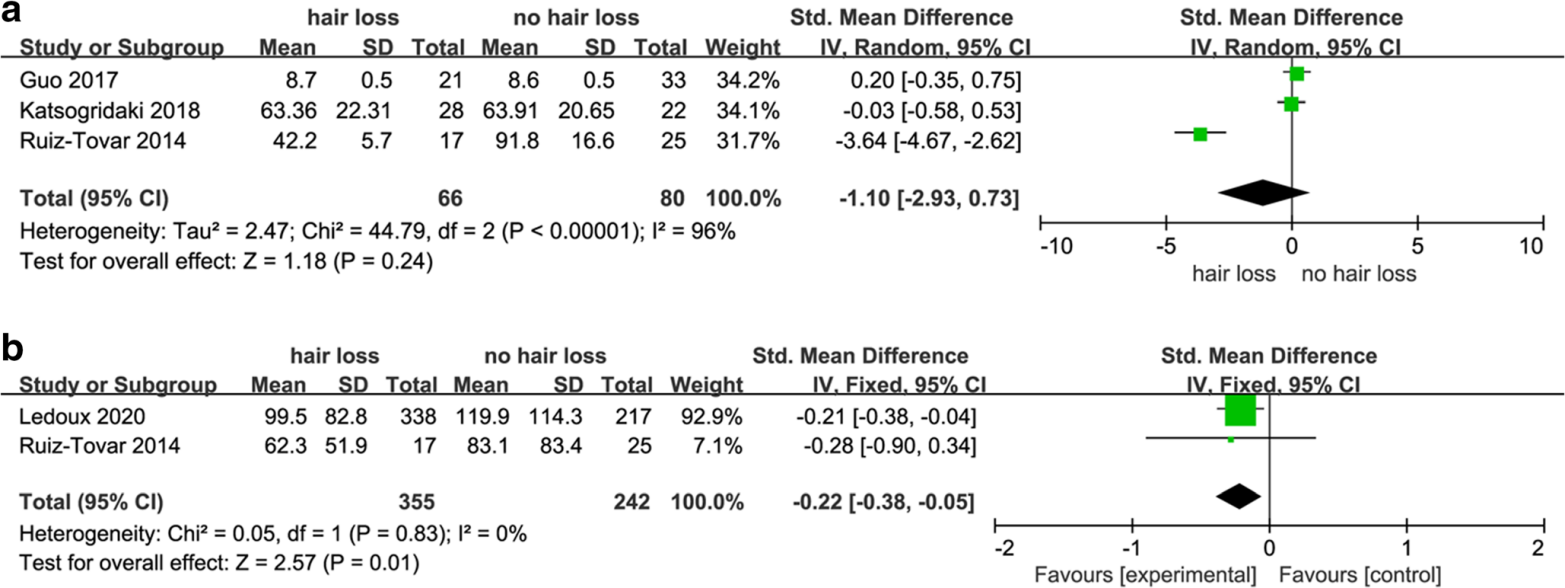
**a** Forest plots of studies in serum iron. **b** Forest plots of studies in serum ferritin

### Iron and Ferritin

For iron, a total of four studies were reviewed (Table 3). Three studies [5, 9, 10] analyzed serum iron levels. Analysis of extracted data showed that there were no significant differences in serum iron levels between patients with or without hair loss after MBS (SMD, − 1.10; 95% CI, − 2.93 to 0.73; *p* = 0.24). The heterogeneity was high *I*^2^ = 96% (Fig. 5a), random effect model was used.

**Table 3:** Studies of iron and ferritin and hair loss

Author (year)	Demographics (hair loss group)	Measures and outcomes
Ledoux 2020 [4]	Sex: 10M/328F Age: 41.5 ± 10.2 Country: France	Mean ferritin levels in hair loss patients (99.5 ± 82.8 μg/l) vs controls (119.9 ± 114.3 μg/l) (*p* < 0.05)
Katsogridaki 2018 [10]	Sex: 5M/23F Age: 38.54 ± 11.04 Country: Greece	No difference in serum iron levels in hair loss patients (63.36 ± 22.31) vs. controls (63.91 ± 20.65) (*p* > 0.05)
Guo 2017 [9]	Sex: 1M/20F Age: 31 ± 6 Country: China	No difference in serum iron levels in hair loss patients (8.7 ± 0.5 mmol/l) vs. controls (8.6 ± 0.5 mmol/l) (*p* > 0.05)
Ruiz-Tovar 2014 [5]	Sex: 0F/16F Country: Spain	Mean iron levels in hair loss patients (42.2 ± 5.7 mg/dL) vs controls (91.8 ± 16.6 mg/dL) (*p* = 0.017) Mean ferritin levels in hair loss patients (62.3 ± 51.9 ng/mL) vs. controls (83.1 ± 83.4 ng/mL) (*p* > 0.05)

**Fig. 6:**
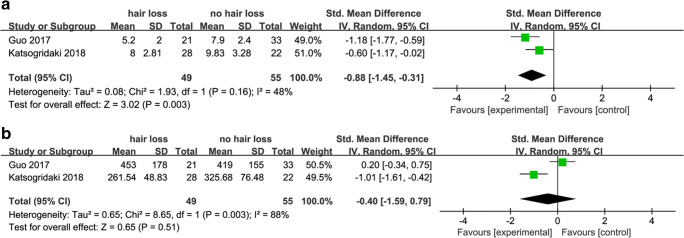
**a** Forest plots of studies in folic acid. **b** Forest plots of studies in vitamin B_12_

Two studies [4, 5] analyzed serum ferritin levels, and both showed lower levels in patients reporting hair loss after MBS (SMD, − 0.22; 95% CI, − 0.38 to − 0.05; *p* = 0.01). Significant heterogeneity was not found *I*^2^ = 0% (Fig. 5b), so a fixed effects model was used.

### Folic Acid and Vitamin B_12_

Studies about the role of folic acid and vitamin B_12_ in hair loss after MBS are summarized in (Table 4). Two of the studies reported the outcome of folic acid levels. Because of heterogeneity among these studies (*p* < 0.0001, *I*^2^ = 48%), a fixed effects model was used to pool results. The result showed that folic acid levels were lower in patients with hair loss (SMD, − 0.88, 95% CI − 1.29 to − 0.46, *p* < 0.0001) (Fig. 6a). The overall analysis showed lower vitamin B_12_ levels in patients reporting hair loss (SMD, − 0.40; 95% CI, − 1.59 to 0.79; *p* = 0.51) (Fig. 6b). The heterogeneity was high *I*^2^ = 88%, a random effects model was used to pool results.

**Table 4:** Studies of folic acid and vitaminB_12_ and hair loss

Author (year)	Demographics (hair loss group)	Measures and outcomes
Katsogridaki 2018 [10]	Sex: 5M/23F Age: 38.54 ± 11.04 Country: Greece	Mean folic acid levels: hair loss (8 ± 2.81 ng/ml) vs. controls (9.83 ± 3.28 ng/ml) (*p* = 0.039) vitamin B_12_: hair loss (261.54 ± 48.83 pg/ml) vs. controls (325.68 ± 76.48 pg/ml) (*p* = 0.001)
Guo 2017 [9]	Sex: 11M/43F Age: 31 Country: China	No difference in vitamin B_12_ levels in hair loss patients (453 ± 178 ng/L) vs. controls (419 ± 155 ng/L) (*p* > 0.05) Mean folic acid levels in hair loss patients (5.2 ± 2.0 μg/L) vs. controls (7.9 ± 2.4 μg/L) (*p* < 0.05)

**Fig. 7:**
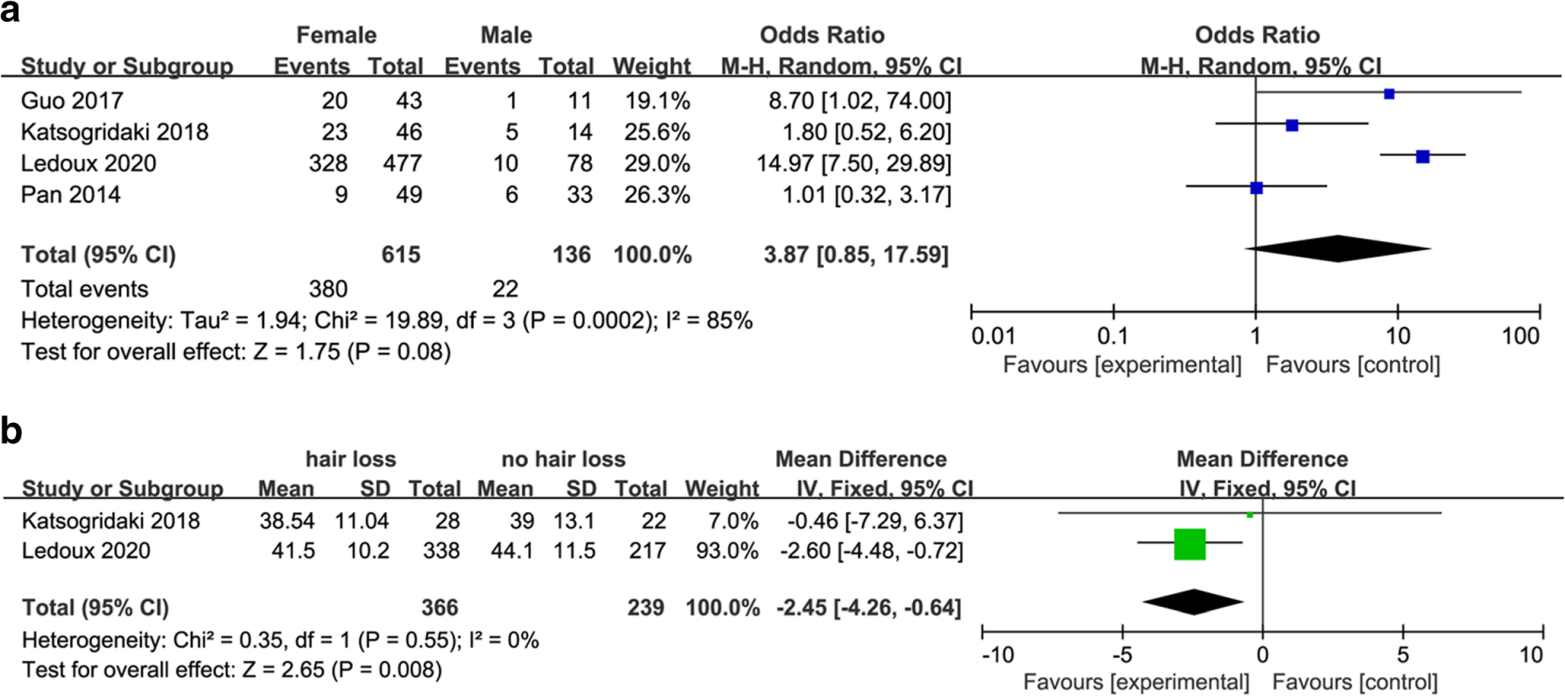
**a** Forest plots of studies in gender. **b** Forest plots of studies in age

### Gender and Age

Studies of gender and age in hair loss after MBS are summarized in (Table 5). Hair loss after MBS was more common in women (OR, 3.87; 95% CI, 0.59 to 17.59; *p* = 0.08; *I*^2^ = 85%) (Fig. 7a), and patients with hair loss were younger than controls (MD, − 2.45; 95% CI, − 4.26 to − 0.64; *p* = 0.008; *I*^2^ = 0) (Fig. 7b).

**Table 5:** Studies of age and gender and hair loss

Author (Year)	Demographics (hair loss group)	Measures and outcomes
Ledoux 2020 [4]	Sex: 10M/328F Age: 41.5 ± 10.2 Country: France	Age (years): hair loss patients ( 41.5 ± 10.2) vs. controls ( 44.1 ± 11.5) (*p* < 0.05) Gender: male (10/78) vs. female with (328/477) (*p* = 0.02)
Katsogridaki 2018 [10]	Sex: 5M/23F Age: 38.54 ± 11.04 Country: Greece	Age: no difference between hair loss patients (38.5 ± 11.04 years) and controls (39 ± 13.1 years) (*p* = 0.892) Gender: male (5/14) vs. female (23/36) (*p* = 0.072)
Guo 2017 [9]	Sex: 11M/43F Age: 31 Country: China	Age: no difference between hair loss patients (31 ± 6 years) and controls (30 ± 7 years)(*p* > 0.05) Gender: male (1/11) vs. female (20/43) (*p* = 0.02)
Pan 2014 [23]	Sex: 6M/9F Age: NR Country: China	NR

*NR* not reported

## Discussion

Hair loss is common after MBS, but there is considerable debate in the scientific literature about its overall incidence and etiopathogenesis. This is the first systematic review in scientific literature on this topic. We found that hair loss happens in 57 % of patients after MBS and that its incidence decreases with longer follow-up times. Serum zinc, ferritin and folic acid levels seem to be lower in patients with hair loss compared with controls. Moreover, young women are more likely to have hair loss. In contrast, evidence remains inconclusive to support any association with low iron or vitamin B_12_ levels.

In this review, there were 18 studies with data regarding hair loss incidence [4, 5, 8–10, 13–25]. From the data, the overall incidence was 57%. The incidence decreased from 58% at less than 12 months follow-up to 35% with more than or equal to 12 months follow-up. This is similar to findings by Guo et al. [9] who observed that hair loss stopped, and new hair gradually grew in all patients after LSG during the follow-up. A retrospective study conducted by Ledoux et al. [4] found that the incidence of hair loss decreases from 65% at < 12 months to 35% at > 12 months follow-up. Most of the weight loss after MBS happens in the first year after surgery [30]. It is possible that the loss of subcutaneous tissue makes it difficult for the scalp to support hair [9].

There were few comparative studies on hair loss after different types of surgeries. Although our results showed that the incidence of hair loss after SG and RYGB was similar, we should treat this result with caution.

We also found that hair loss after MBS was more common in women and patients with hair loss were younger than controls. Researchers have proposed that women have longer hair than men and have higher requirements for scalp support [9]. Moreover, younger women may report hair loss more frequently, from an esthetic perspective [4]. This might indicate a special need for attention in younger women after surgery.

Our meta-analysis found zinc deficiency to be associated with hair loss after MBS (*p* = 0.05) which is consistent with other reviews and meta-analyses showing an association between alopecia and zinc levels [31–33]. Though it may not be cost-effective to screen for and treat all zinc deficiency after MBS, one should ensure that patients, particularly those undergoing a gastric bypass are on multivitamin tablets that provide at least 30 mg of zinc daily [34]. Patients with significant hair loss may benefit from higher dosages of zinc supplementation.

Iron deficiency is a common occurrence after MBS but its relationship with hair loss after MBS is controversial [35–41]. In this review, serum ferritin levels were significantly associated with hair loss following MBS, but serum iron levels were not. Serum ferritin levels reflect a patient’s total iron storage [42]. However, studies have shown that serum ferritin levels may be altered with infectious, inflammatory, and neoplastic conditions [42]. That is why we have concluded that there is currently insufficient evidence to claim a role for iron deficiency as a causative factor for hair loss after MBS.

There is a paucity of data on folic acid and vitamin B_12_ levels in patients with and without hair loss following MBS in the published literature. Only two studies presented data on folic acid and vitamin B_12_ levels [9, 10]. Results suggested that there were significant differences in folic acid levels between both groups. Further analysis showed that vitamin B_12_ was not associated with hair loss. Because of the low number of eligible studies, this finding has to be treated with caution and further studies are necessary before we can reach any conclusion.

In addition to the above, Ledoux et al. [4] found that, in subjects with hair loss, blood parameters of protein were significantly lower than in subjects without hair loss. Postoperative excess weight loss (EWL) was found to be a significant factor in the study by Guo et al. [9] Moreover, acute stress, such as surgical trauma, catabolic state after bariatric surgery, and psychological stress due to factors such as stigma and discrimination associated with obesity and surgery [43], may inhibit hair growth through the release of cortisol and substance P-mast cell pathway. The stress hormone cortisol reduces synthesis and speeds up the degradation of important skin components [44]. All these factors can also be hypothesized to play a role in the etio-pathogenesis of hair loss after MBS.

### Strengths and Limitations

To our knowledge, this is the first systematic review and meta-analysis on hair loss after bariatric surgery.

Our review has some limitations. Firstly, the number of included studies on the effects of hair loss is limited, which may affect the reliability of the results. More studies are needed on this topic. Moreover, reporting of hair loss by patients can be very subjective. There is currently a lack of standardized criteria for the diagnosis of this condition.

Lastly, most of the included studies were retrospective observational studies, which means our findings need further confirmation in higher quality studies. Randomized studies are needed.

## Conclusions

Approximately 57.0% of patients experience hair loss after metabolic and bariatric surgery. Younger age, female sex, low folic acid levels, low zinc levels, and low ferritin levels were associated with it. Our findings could prove helpful in the diagnosis and treatment of these patients. Larger, randomized studies are needed.
